# Prognostic role of noninvasive liver reserve markers in patients with hepatocellular carcinoma undergoing transarterial chemoembolization

**DOI:** 10.1371/journal.pone.0180408

**Published:** 2017-07-03

**Authors:** Shu-Yein Ho, Po-Hong Liu, Chia-Yang Hsu, Cheng-Yuan Hsia, Yun-Hsuan Lee, Rheun-Chuan Lee, Yi-Hsiang Huang, Fa-Yauh Lee, Ming-Chih Hou, Ya-Ju Tsai, Teh-Ia Huo

**Affiliations:** 1 Department of Medicine, Taipei Veterans General Hospital, Taipei, Taiwan; 2 Faculty of Medicine, National Yang-Ming University School of Medicine, Taipei, Taiwan; 3 Harvard T.H. Chan School of Public Health, Boston, Massachusetts, United States of America; 4 Department of Internal Medicine, University of Nevada School of Medicine, Reno, Nevada, United States of America; 5 Department of Surgery, Taipei Veterans General Hospital, Taipei, Taiwan; 6 Department of Radiology, Taipei Veterans General Hospital, Taipei, Taiwan; 7 Institute of Clinical Medicine, National Yang-Ming University School of Medicine, Taipei, Taiwan; 8 Renown Regional Medical Center, Reno, Nevada, United States of America; 9 Institute of Pharmacology, National Yang-Ming University School of Medicine, Taipei, Taiwan; Chang Gung Memorial Hospital Kaohsiung Branch, TAIWAN

## Abstract

**Background:**

Various noninvasive liver reserve markers were proposed to indicate the severity of liver damage. However, the role and feasibility of these markers to predict the prognosis of patients with hepatocellular carcinoma (HCC) are unknown. We aimed to identify the prognostic role of the 8 currently used hepatic reserve markers in patients with HCC undergoing transarterial chemoembolization (TACE).

**Methods:**

Between 2002 and 2013, a total of 881 patients with HCC undergoing TACE were prospectively identified and retrospectively analyzed. The baseline characteristics, tumor status and noninvasive markers were collected. Homogeneity and corrected Akaike information criteria (AICc) were compared between these markers. The Cox proportional hazards model was used to identify independent predictors of survival.

**Results:**

Significant differences in survival distribution were found for albumin-bilirubin (ALBI) grade, Child-Turcotte-Pugh (CTP) class, Lok index, fibrosis index based on 4 factors (FIB-4), Göteborg University cirrhosis index (GUCI), cirrhosis discriminant index (CDI) and model for end-stage liver disease (MELD) score (all p values <0.05). Among these markers, the ALBI grade showed the highest homogeneity and lowest AICc value, indicating a better prognostic performance. Cox multivariate analysis confirmed that ALBI grade 2, ascites, serum alkaline phosphatase and α-fetoprotein level, tumor diameter, vascular invasion and performance status were significant independent prognostic predictors. The distribution of the ALBI score well correlated with baseline CTP and MLED scores.

**Conclusions:**

Our data suggest that among the currently used liver reserve markers, ALBI grade may serve as an objective and feasible surrogate to predict the prognosis of HCC patients undergoing TACE.

## Introduction

Hepatocellular carcinoma (HCC) is the sixth most common malignancy and the third leading cause of cancer-related mortality worldwide [1]. The incidence of HCC is highest in Southeast Asia and sub-Saharan Africa where hepatitis B virus (HBV) infection is endemic. Hepatitis C virus (HCV)-associated HCC increased rapidly in United State [2, 3]. HCC typically develops on a background of chronic liver disease or cirrhosis in 70–90% of all cases [4, 5]. As a result, various degrees of liver functional insufficiency are usually present at the time of cancer diagnosis. Surgical resection is generally recommended for HCC [6], but is indicated only for patients with early stage and well preserved liver function. For patients not suitable for curative treatment, transarterial chemoembolization (TACE) may provide better loco-regional tumor control and increase patient survival [7].

In comparison with other solid cancers, management and prognosis HCC highly depend on tumor extent and underlying liver functional reserve [8]. The Child-Turcotte-Pugh (CTP) classification is widely used to evaluate liver reserve in patients with HCC. However, it has some limitations because the cut-off values of some variables (serum albumin, bilirubin and international normalized ratio of prothrombin time) are arbitrarily defined; in addition, ascites and encephalopathy are highly subjective [9]. Although liver biopsy is usually the gold standard to assess hepatic fibrosis or cirrhosis, it is an invasive procedure that is associated with potential risks and prone to sampling errors as well as intra- and inter-observer variation [10].

Up to now, at least 8 noninvasive liver reserve markers have been proposed to define the degree of functional liver reserve in patients with chronic liver diseases [11]. The model for end-stage liver disease (MELD) has been adopted for end-stage cirrhotic patients waiting for liver transplantation and has been used to assess liver dysfunction in HCC [12]. Alternative tools to evaluate liver dysfunction are Lok index, cirrhosis discriminant index (CDS) and Göteborg University Cirrhosis Index (GUCI) [13–15]. These markers incorporate clinical parameters such as serum aspartate aminotransferase, alanine aminotransferase, international normalized ratio of prothrombin time (INR) and platelet count. Other liver reserve markers, fibrosis index based on 4 factors (FIB-4) and aspartate aminotransferase-to-platelet ratio (APRI), have also been proposed to assess liver dysfunction [16, 17]. Both FIB-4 index and APRI can be readily calculated by using clinical variables including age and serum biochemistry. Lastly, the albumin-bilirubin (ALBI) grade is a more recently introduced prognostic marker based solely on serum albumin and bilirubin level [18].

Given all these choices, their role and accuracy in predicting the outcome of HCC patients are largely unclear. Selection of an optimal surrogate marker for these patients is highly controversial. This study aimed to assess the feasibility and compare the prognostic role of these markers in patients with HCC undergoing TACE.

## Methods

### Patients

Between 2002 and 2013, patients with HCC undergoing TACE in Taipei Veterans General Hospitals were prospectively collected and retrospectively analyzed. The baseline characteristics, including demographic data, etiology of chronic liver diseases, performance status, diabetes mellitus, laboratory parameters, tumor status (tumor nodules, tumor diameter and vascular invasion) and various noninvasive markers for liver reserve, were comprehensively recorded at the time when the diagnosis was established. Patients were followed every 3–6 months until death or dropout from follow-up. This study was approved by the Institutional Review Broad of Taipei Veterans General Hospital (IRB protocol number 2014-03-007AC), and complies with the standards of the Declaration of Helsinki and current ethical guidelines. Waiver of consent was obtained, and patient records/information was anonymized and de-identified prior to analysis.

### Diagnosis and definition

The diagnosis of HCC was histologically confirmed by needle biopsy or based on the findings of typical radiological features in at least two imaging examinations including sonography, contrast-enhanced dynamic computed tomography (CT), magnetic resonance imaging (MRI), and hepatic arterial angiography [19]. Patients who were seropositive for hepatitis B antigen (HBsAg), seronegative for anti-HCV antibody, and without history of alcoholism were classified as HBV-related HCC. HCV-related HCC was defined as seropositive for anti-HCV antibody, seronegative for HBsAg and no history of alcoholism. Dual HBV- and HCV-related HCC was defined as seropositive for HBsAg and anti-HCV antibody [20]. The 8 liver reserve markers were calculated according to their original formula, and grading of severity was classified at the time of diagnosis according to the scores [15, 21, 22].

### Treatment

When the diagnosis of HCC was confirmed, patients’ medical data were reviewed at the multidisciplinary HCC broad of Taipei Veterans General Hospital. The management of unresectable HCC and indication of TACE were according to the American Association for the Study of Liver Disease or European Association for the Study of Liver guidelines [23]. TACE was performed in patients who had unresectable lesions and were not eligible or unwilling to receive other therapies. The HCC nodule(s) was considered unresectable if there were multifocal lesions which made extended resection necessary to eradicate all tumors, or hepatic reserve was insufficient with an indocyanine green 15-min retention rate > 30%. The criteria for patients undergoing TACE were: (1) no main portal vein trunk involvement or extrahepatic metastasis, (2) CTP functional class A or B, (3) normal renal function with a serum creatinine concentration < 1.5 mg/dL, and (4) no gross ascites by ultrasound or CT [24, 25]. Therapeutic information including benefits and risks was provided to individual patient based on shared decision making. Written informed consent was obtained prior to initiation of treatment.

The Seldinger’s technique of arterial embolization was administered as standard TACE procedure [23–25]. In brief, the four-French catheter (Terumo, Tokyo, Japan) were used for femoral artery puncture. HCC nodules were localized with hepatic arteriography and superior mesenteric arterial portovenography. Tumor stain (vascularity of HCC tumor) was investigated with 50 to 100 mL radiocontrast agent (Telebrix; Laboratoire Guerbet, Aulnay-Sous-Bois, France) injected by a power injector (CT9000 ADV; Liebel-Flarsheim, St. Louis, MO, USA). Infusion of a mixture of 20–30 mg adriamycin (Carlo Erba, Milan, Italy) and 5–10 mL Lipiodol (Laboratoire Guerbet) was performed after the arteries supplying the tumor were catheterized with three-French catheter superselectively. Sufficient amount of emulsion to the tumoral area and 2–3 mm strips of Gelfoam (Upjohn, Kalamazoo, MI, USA) were delivered till complete flow stagnation was achieved.

### Statistics

The two-tailed chi-squared or Fisher exact test was employed to compare categorical data. The Mann-Whitney rank sum test was used for continuous variables. Missing values were handled by multiple imputation while a complete case was used as benchmark analysis. Logistic regression on missing data indicators using completely observed variables as covariates was implemented. The statistical output obtained from multiple imputation was similar to statistical output from a complete case analysis in which patients with missing data were omitted. Pooled results by multiple imputation were reported in this study [26, 27]. The survival distributions for liver reserve markers were examined by the Kaplan-Meier methods and compared by log-rank test. Comparison of prognostic performance of these markers was calculated by corrected Akaike information criteria (AICc) and homogeneity. Prognostic factors that were possibly linked to survival, including sex, etiology of chronic liver disease, performance status, laboratory parameters and tumor status were comprehensively included in survival analysis. Factors that were significant in the univariate analysis were introduced into the multivariate Cox proportional hazards model to determine the adjusted risk ratio. Box plot was used to describe correlation between ALBI grade and CTP and MELD grades. All statistical analyses were conducted using the SPSS for Windows version 21 release (SPSS Inc., Chicago, IL, USA). Statistical significance was set at *p* value < 0.05 in two-tailed tests.

## Results

A total of 881 patients who received TACE as primary anti-cancer treatment were enrolled between 2002 and 2013. The median age was 68 years and 673 (76%) patients were male. The median overall survival of the study patients was 24 months. The etiology of HCC were hepatitis B in 311 (35%), hepatitis C in 241 (27%), both hepatitis B and C in 35 (4%), and alcoholism in 162 (19%) patients. There were 166 (18%) patients who had ascites at the time of diagnosis and 226 (26%) patients had history of diabetes mellitus. Four hundred and forty-two (50%) patients had a single tumor at initial presentation and 450 (51%) patients had maximum tumor diameter < 5 cm (Table 1). The calculation formula and severity grading of the 8 liver reserve markers are described in Table 2.

**Table 1:** Baseline characteristics of patients of hepatocellular carcinoma undergoing TACE.

Variables	Patients (n = 881)
Age (years, median [interquartile range])	68 (55–75)
Male, n (%)	673 (76)
Etiologies of liver disease	
HBV, n (%)	311 (35)
HCV, n (%)	241 (27)
HBV+HCV, n (%)	35 (4)
Alcohol, n (%)	162 (19)
Diabetes mellitus, n (%)	226 (26)
Performance status (0/1/2/3/4), n (%)	521/207/106/39/8 (59/24/12/4/1)
Ascites, n (%)	166 (18)
Laboratory values	
α-fetoprotein (ng/mL), mean ± SD	13515.0 ± 98741.32
Alanine aminotransferase (IU/L), mean± SD	67.2±70.1
Aspartate aminotransferase (IU/L), mean± SD	94.2± 184.1
Alkaline phosphatase (IU/L), mean± SD	138.62±103.77
Albumin (g/L), mean ± SD	36± 6
Total bilirubin (μmol/L), mean ± SD	18.8 ± 16.6
Creatinine (mg/dl), mean ± SD	1.151±0.956
Platelets (1,000/μL), mean ± SD	160± 95
INR of prothrombin time (mean ± SD)	1.1 ± 0.2
Non-invasive liver reserve markers	
ALBI grade (1/2/3), n (%)	297/540/44 (34/61/5)
APRI grade (1/2/3), n (%)	195/368/318 (22/42/36)
CTP classification (A/B/C), n (%)	698/263/20 (79/19/2)
CDS grade (1/2/3), n (%)	209/500/172 (24/56/20)
FIB-4 grade (1/2/3), n (%)	401/234/246 (45/27/28)
GUCI grade (1/2/3), n (%)	154/362/365 (18/41/41)
Lok index grade (1/2/3), n (%)	365/298/218 (41/34/25)
MELD score (<10/10-14/>14), n (%)	613/202/66 (70/23/7)
Tumor nodules (1/2/≥3), n (%)	442/177/262 (50/20/30)
Maximal tumor diameter (≤2/ 2-5/ >5cm), n (%)	121/329/431 (14/37/49)
Vascular invasion, n (%)	157 (18%)

**Table 2:** Formula and grading of the 8 noninvasive hepatic reserve markers.

Noninvasive blood tests as liver reserve markers	Formula
ALBI, Grade 1/2/3(<-2.6/-2.6- ≤-1.39 / >-1.39)	(log(bilirubin[μmol/L]) x 0.66)—(albumin[g/L] x 0.085)
APRI, Grade 1/2/3(<0.5/0.5–1.5/ >1.5)	AST (/UNL)/platelet (109/L) × 100
CTP, A/B/C, grade 1/2/3/(5-6/7-9/10-15)	encephalopathy: none = 1, grade 1 or 2 = 2, grade 3 or 4 = 3 ascites: none = 1, mild to moderate = 2, severe = 3 bilirubin (mg/dl): <2 = 1, 2–3 = 2, >3 = 3albumin (g/dl): >3.5 = 1, 2.8–3.5 = 2, < 2.8 = 3PT sec (INR): < 4 (1.7) = 1, 4–6 (1.7–2.3) = 2, > 6 (>2.3) = 3
CDS, Grade 1/2/3(<4/4-7/>7)	platelet count (× 10^9^/L): >340 = 0; 280–339 = 1; 220–279 = 2; 160–219 = 3; 100–159 = 4; 40–99 = 5; <40 = 6
	ALT/AST ratio: >1.7 = 0; 1.2–1.7 = 1; 0.6–1.19 = 2; <0.6 = 3
	INR: <1.1 = 0; 1.1–1.4 = 1; >1.4 = 2; CDS is the sum of the above (possible value 0–11)
FIB-4 index, Grade 1/2/3(<1.45/1.45–3.25/>3.25)	Age × AST/[Platelet × (ALT)^1/2^]
GUCI, Grade 1/2/3(<0.5/0.5–1.56/>1.56)	AST/TOP NORMAL AST x INR x100/(Platelets x10^9^)
Lok index, Grade 1/2/3(<0.5/0.5–0.8/>0.8)	Lok Index = e^(LogOddsLok)^ / (1 + e^(LogOddsLok)^)Log Odds Lok = (1.26x AST / ALT) + (5.27 x INR)—(0.0089 x Platelets x10^9^)—5.56
MELD, Grade 1/2/3(<10/10-14/>14)	10 x ((0.957 x ln(Creatinine)) + (0.378 x ln(Bilirubin)) + (1.12 x ln(INR))) + 6.43

**Fig 1:**
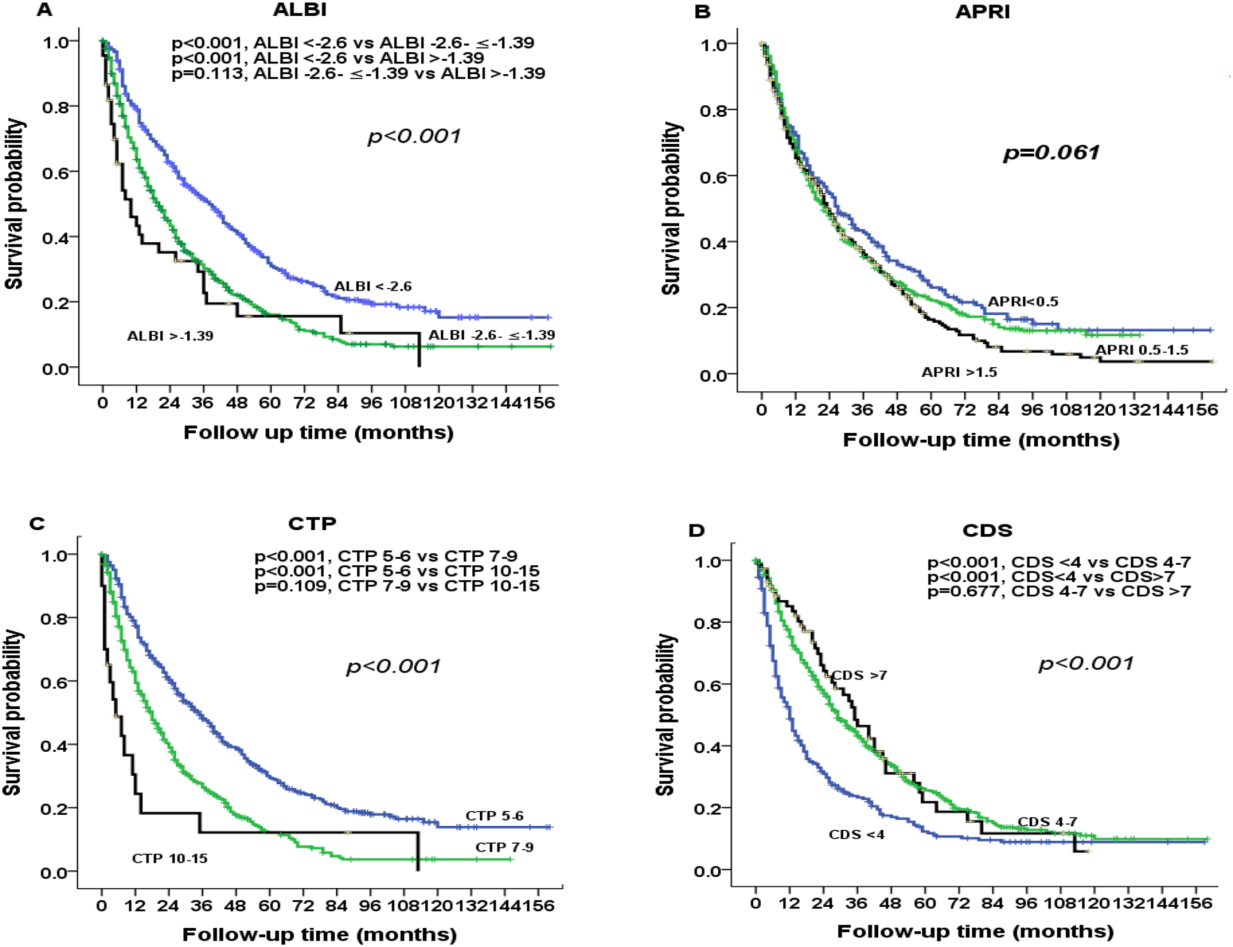
Comparison of survival distributions according to (A) ALBI, (B) APRI, (C) CTP, and (D) CDS grading. Significant survival differences are found for ALBI grade, CTP class and CDS grading.

**Fig 2:**
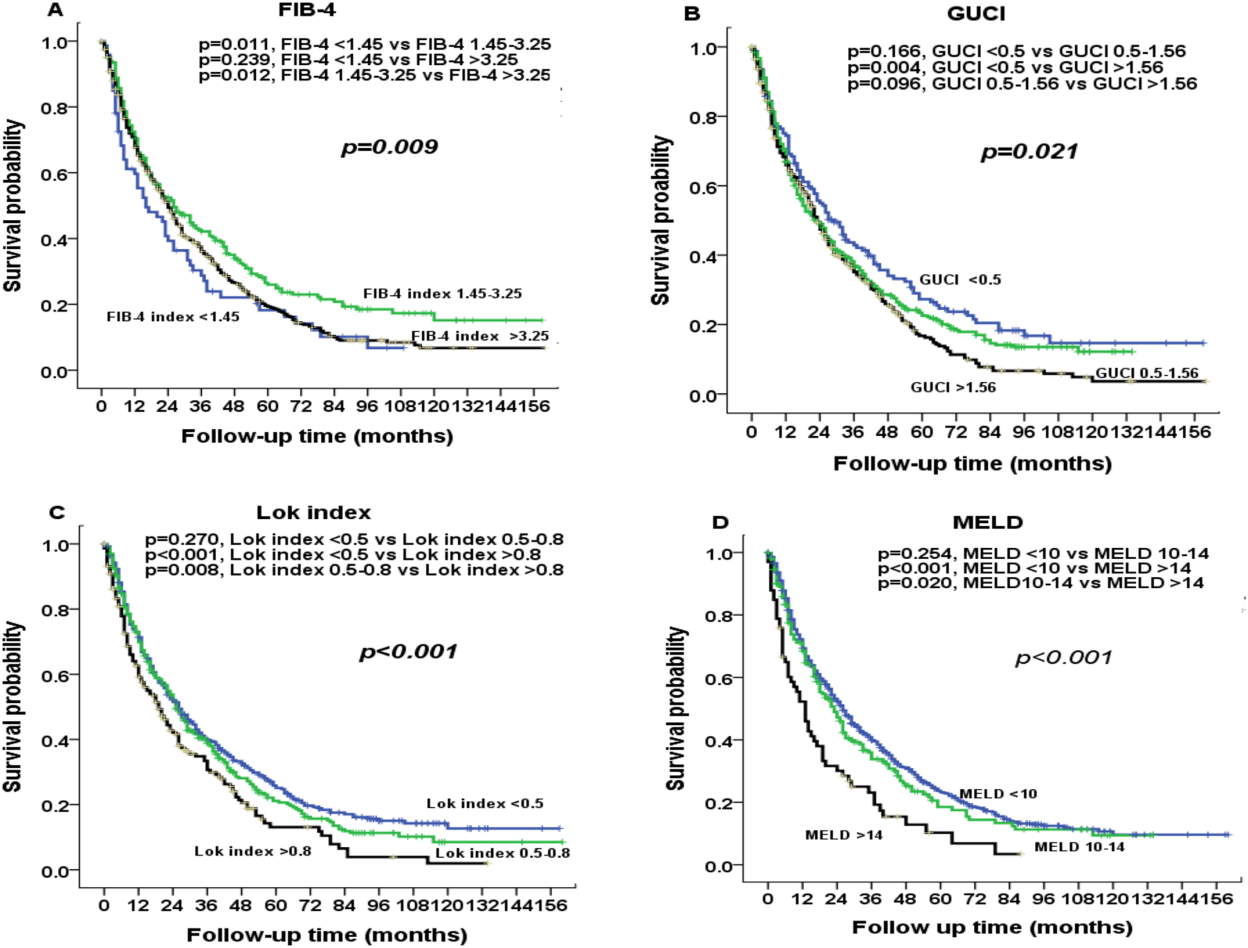
Comparison of survival distributions according to (A) FIB-4 index, (B) GUCI, (C) Lok index, and (D) MELD grading. Significant survival differences are found in all 4 markers.

**Fig 3:**
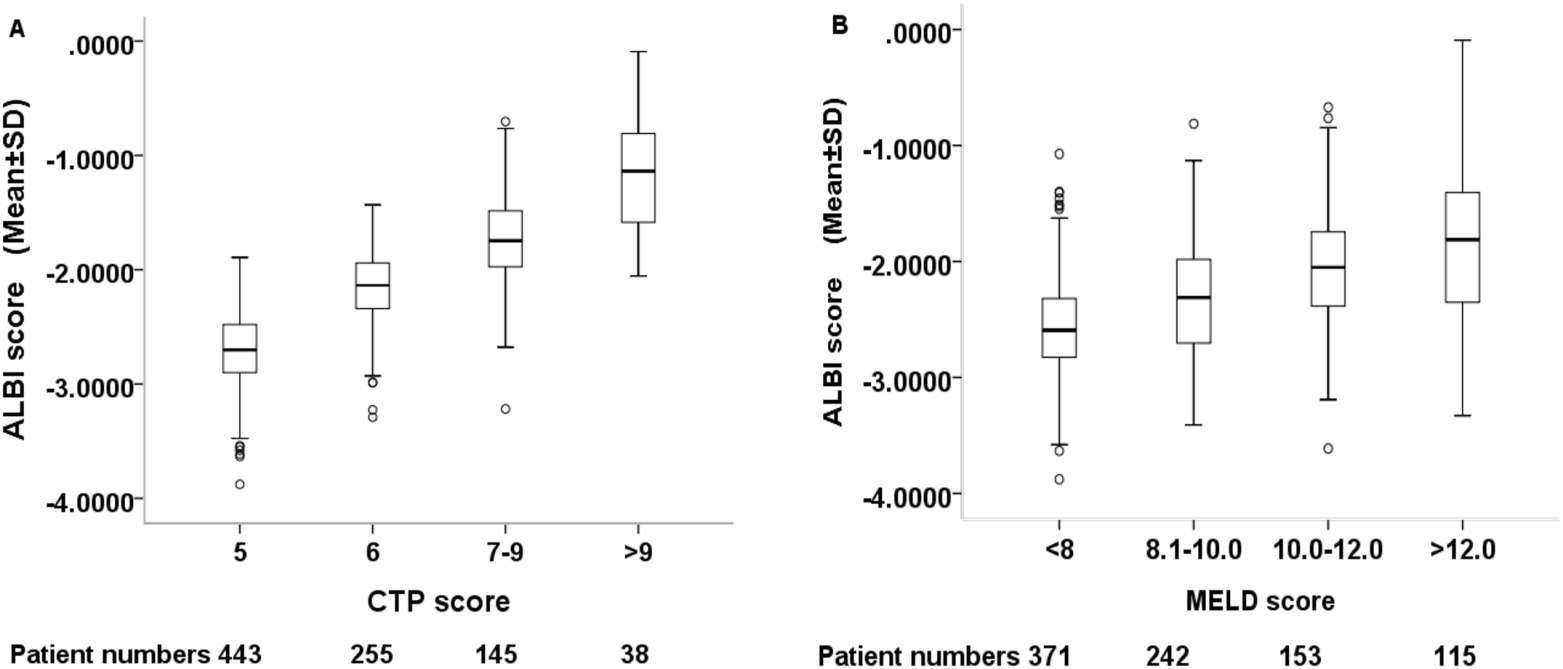
Correlation between ALBI score with CTP score and MELD score. The ALBI score increases with increasing CTP and MELD scores. The dark line in the middle of the boxes is the median of ALBI score. The bottom of the box indicates the 25th percentile and the top of the box represents the 75th percentile. T-bar at the top and bottom of the box is maximum and minimum values, respectively. ○ indicates extreme values. SD; standard deviation.

The prognostic value of these markers was evaluated according to their grading (Figs 1 and 2). Significant differences in survival distribution were found in all markers with the exception of APRI score. Pairwise comparison showed that there were no significance survival differences between ALBI grade 2 vs 3 (*p* = 0.113), CTP grade 2 vs 3 (*p* = 0.109), CDS grade 2 vs 3 (*p* = 0.677), FIB-4 grade 1 vs 3 (*p* = 0.239), GUCI grade 1 vs 2 (*p* = 0.166), GUCI grade 2 vs 3 (*p* = 0.096), Lok index grade 1 vs 2 (*p* = 0.270) and MELD grade 1 vs 2 (*p* = 0.254). Fig 3 shows the correlation between the ALBI score and baseline CTP and MLED scores. The ALBI score increased with increasing CTP and MELD scores, indicating a worsened liver functional reserve.

Comparison of the prognostic performance of the 8 markers is shown in Table 3. Of these, the ALBI grade had the highest homogeneity and lowest AICc value, followed by the CDS and CTP class. The predictive role of the ALBI score was analyzed along with other clinically important prognostic predictors. In univariate survival analysis, alcoholism, presence of ascites, alkaline phosphatase level, serum platelet count and AFP level, maximum tumor diameter, vascular invasion, performance status and ALBI grade were the factors associated with a poor prognosis (Table 4; all *p* values <0.05). Cox multivariate analysis revealed that ascites [hazard ratio (HR): 1.536, 95% confidence interval (CI): 1.227–1.924, *p*< 0.001], alkaline phosphatase ≧100 IU/L [HR:1.362, 95% CI: 1.155–1.607, *p*< 0.001], AFP ≧20 ng/dL [HR: 2.006, 95% CI: 1.694–2.372, *p*< 0.001], maximum tumor diameter > 5 cm [HR:1.791, 95% CI: 1.510–1.2.124, *p*< 0.001], vascular invasion [HR: 1.999, 95% CI: 1.622–2.646, *p*< 0.001], poor performance status [HR: 1.463, 95% CI: 1.221–1.751, *p*< 0.001], and ALBI grade 2 [HR: 1.531, 95% CI: 1.285–2.823, *p*< 0.001] were independent predictors associated with a decreased survival; ALBI grade 3 was associated with a decreased survival at marginal significance [HR: 1.525, 95% CI: 0.967–2.38, *p* = 0.064] in the Cox model.

**Table 3:** Comparison of prognostic performance of noninvasive liver reserve markers.

	Homogeneity (Wald χ2)	Corrected Akaike information criteria (AICc)
ALBI	43.655	8094.296
APRI	2.050	8135.901
CTP	26.861	8111.090
CDS	35.635	8102.143
FIB-4	0.173	8137.571
GUCI	7.613	8130.338
Lok index	11.512	8126.439
MELD	6.700	8131.251

ALBI, albumin-bilirubin; APRI, aspartate transaminase-to-platelet ratio; CDS, cirrhosis discriminant index; CTP, Child-Turcotte-Pugh score; FIB-4, fibrosis index based on the four factors (FIB-4); MELD, model for end-stage liver disease; GUCI, Göteborg University Cirrhosis Index.

**Table 4:** Univariate and multivariate survival analysis in patients with hepatocellular carcinoma undergoing TACE.

Overall survival	Number	Univariate analysis			Multivariate analysis		
		HR	CI	*p*	HR	CI	*p*
§All patients (n = 881)							
Age (<65/≥65 years)	373/508	0.933	0.801–1.088	0.377			
Sex (male/female)	673/208	0.936	0.855–1.025	0.153			
HBsAg (negative/positive)	466/415	1.1271	0.969–1.311	0.120			
Anti-HCV (negative/positive)	572/309	0.894	0.764–1.048	0.167			
Alcoholism (no/yes)	719/162	1.240	1.018–1.509	0.032			
^a^Diabetes mellitus (no/yes)	651/226	1.041	0.873–1.240	0.656			
Ascites (absent/present)	715/166	1.796.522	1.488–2.169	<0.001	1.536	1.227–1.924	<0.001
Creatinine (<1/≥1 mg/dL)	442/439	0.897	0.772–1.043	0.159			
Alanine transaminase (≤40/>40 IU/L)	337/544	1.151	0.985–1.346	0.077			
^a^Alkaline phosphatase (<100/≥100 IU/L) 1.06	370/492	1.678	1.435–1.963	<0.001	1.362	1.155–1.607	<0.001
INR of PT (<1/≥1)	236/645	1.145	0.967–1.354	0.116			
Platelet (≥150,000/<150,000/μL)	408/473	0.794	0.683–0.924	0.003			
Alpha-fetoprotein (<20/≥20 ng/mL)	349/532	1.927	1.642–2.261	<0.001	2.006	1.694–2.372	<0.001
Tumor nodules (single/multiple)	442/439	1.152	0.990–1.339	0.067			
Maximal tumor diameter (≤5/>5 cm)	450/431	2.031	1.743–2.366	<0.001	1.791	1.510–2.124	<0.001
Vascular invasion (no/yes)	724/157	2.511	2.075–3.039	<0.001	1.999	1.622–2.464	<0.001
Performance status (0/1-4)	521/360	1.779	1.521–2.081	<0.001	1.463	1.221–1.751	<0.001
Albumin-Bilirubin grade							
Grade 1	297	1					
Grade 2	540	1.678	1.421–1.981	<0.001	1.531	1.285–2.823	<0.001
Grade 3	44	1.501	1.251–1.801	<0.001	1.525	0.976–2.382	0.064

HR, hazards ratio; CI, confidence interval; HCV, hepatitis C virus, INR, international normalized ratio; PT, prothrombin time

^a^: Missing data of DM and alkaline phosphatase in 4 (0.5%) and 19 (2.2%) patients, respectively.

## Discussion

Liver functional reserve is a critical concern in determining the prognosis of HCC. Efforts were undertaken to identify accurate surrogate markers to indicate the severity of liver dysfunction in HCC patients during the past decades [6, 28]. In this study, we enrolled a large, well-documented, and adequately followed-up HCC cohort. Up to 8 noninvasive liver reserve markers and possible prognostic predictors were comprehensively evaluated. We confirm that the key prognostic predictors of HCC are the severity of liver reserve, tumor burden and performance status of patients. We also show that among the 8 noninvasive markers, the ALBI grade is the best predictive model to assess the degree of liver damage in HCC patients undergoing TACE. These data, which are consistent with previous cohort studies [18, 22, 29], can discriminate patient survival and indicate the predictive accuracy of the ALBI grade for HCC patients.

With Kaplan-Meier survival analyses, we systematically investigated 8 noninvasive liver reserve markers in HCC patients undergoing TACE. Our results show that ALBI, CDS and CTP are the three most accurate prognostic markers to distinguish hepatic functional reserve according to the AICc analysis. Of these markers, we found that the ALBI model is the best in discriminating survival for different severity grades. In addition, the ALBI has the greatest homogeneity of survival among patients within the same stage, suggesting ALBI is a more feasible tool for outcome prediction among the 8 markers. In multivariate Cox analysis, patients with ALBI grade 2 and grade 3 had 52–53% increased risk of mortality as compared with patients with ALBI grade 1. These findings indicate that ALBI grade is superior in representing liver reserve and providing prognostic information.

In this study, we aim to determine the independent prognostic predictors for HCC. In additional to ALBI grade, we found that the number and size of tumor nodule were closely related to overall survival of HCC patients. Additionally, consistent with published data [29–33], vascular invasion was identified as an important predictor for patient survival. The performance status of patient, as determined according to the Eastern Cooperative Oncology Group scale, may also independently influence the survival in HCC patients [34, 35]. Moreover, in accordance with previous studies [26, 32, 35–37], our study found that presence of ascites, high AFP and serum alkaline phosphatase level were strongly linked with patient outcomes. Taken together, the extent of tumor involvement, performance status and severity of liver reserve are the hallmarks of survival predictors.

The CTP, MELD and ALBI grades are three major composite models to assess the severity of liver functional reserve. A major shortcoming of the CTP score is its arbitrary cut-off values and subjective variables such as encephalopathy [9]. MELD score is an alternative commonly used marker to indicate liver dysfunction in HCC, and may perform better than the CTP score in different clinical settings. However, serum creatinine level, which is one of the parameters in the MELD, may be less reliable in patients with HCC because cancer-related cachexia might not be fully reflected. Notably, the prognostic role of the other 5 models (Lok index, FIB-4, APRI, GUCI and CDS) has never been validated in patients with HCC undergoing different therapies. In comparison with the other 7 models, the ALBI grade, which incorporates only serum albumin and bilirubin level, is more objective and can be rapidly computed without the need for other special tests [18, 22, 29, 38, 39]. Our study suggests that the ALBI grade is a more feasible and readily available liver reserve marker for risk stratification in HCC patients undergoing TACE.

Among the 8 markers, APRI and FIB-4 index were principally designed as liver fibrosis markers. Therefore, it should be noted that the prognostic impact of these markers could be through the high hepato-carcinogenic potential in the background liver which is associated with increased risk of tumor recurrence, and may not be necessarily through the deterioration of liver functional reserve.

The ALBI score well correlated with baseline CTP and MELD score in HCC patients, suggesting these three models are intrinsically similar tools in assessing liver functional reserve. However, the latter two models performed less well in the cohort of HCC patients undergoing TACE. These results imply that the evaluation based on albumin and bilirubin is clinically more robust and accurate, and the possibility that the ALBI grade may be integrated into the cancer staging system to further refine prognostic information should be considered. However, a potential weakness of the ALBI score is that it could be influenced by albumin replacement therapy or the presence of obstructive jaundice when in some cases HCC may present with obstructive jaundice, and thus might not accurately reflect true liver functional reserve at all times.

This study has a few limitations. First, this is a single center, retrospective study in a predominantly HBV endemic area. Our results require external validation from independent research groups. Second, this study is limited to HCC patients undergoing TACE to specifically address the prognostic role of different liver reserve markers. The accuracy of ALBI grade in patients receiving other therapies needs further studies to establish. Third, some patients did not strictly adhere to the Barcelona Clinic Liver Cancer (BCLC) recommendations. Rather, treatment decisions were decided by the patients and the multidisciplinary HCC team based on shared decision making.

In conclusion, our results indicate that the ALBI grade is the most accurate prognostic model among the 8 noninvasive liver reserve markers. The ALBI grade may serve as an objective, discriminatory and evidence-based method in assessing liver functional reserve. The ALBI grade is clinically more useful due to is superior prognostic power in HCC patients undergoing TACE. Further studies are urgently needed to validate the feasibility of ALBI grade in different clinical scenarios.

## Abbreviations

AFP: α-fetoprotein
ALBI: albumin-bilirubin
APRI: aspartate aminotransferase-to-platelet ratio
BCLC: Barcelona Clinic Liver Cancer
CDS: cirrhosis discriminant index
CI: confidence interval
CT: computed tomography
CTP: Child-Turcotte-Pugh
FIB-4: fibrosis index based on 4 factors
GUCI: Göteborg University cirrhosis index
HBV: hepatitis B virus
HCC: hepatocellular carcinoma
HCV: hepatitis C virus
INR: international normalized ratio
MELD: model for end-stage liver disease
MRI: magnetic resonance imaging
SD: standard deviation
TACE: transarterial chemoembolization
